# Evaluating disparities by social determinants in hospital admission decisions for patients with COVID-19 quaternary hospital early in the pandemic

**DOI:** 10.1097/MD.0000000000033178

**Published:** 2023-03-10

**Authors:** Peter K. Olds, Nicholas Musinguzi, Benjamin P. Geisler, Jessica E. Haberer

**Affiliations:** a Massachusetts General Hospital, Harvard Medical School, Boston, MA; b Mbarara University of Science and Technology, Mbarara, Uganda; c Ludwig Maximilian University Munich, Munich, Germany.

**Keywords:** admission practices, COVID-19, disparities, hospital, social determinants of health

## Abstract

The COVID-19 pandemic has highlighted significant disparities in hospital outcomes when focusing on social determinants of health. Better understanding the drivers of these disparities is not only critical for COVID-19 care but also to ensure equitable treatment more generally. In this paper, we look at how hospital admission patterns, both to the medical ward and the intensive care unit (ICU), may have differed by race, ethnicity, and social determinants of health. We conducted a retrospective chart review of all patients who presented to the Emergency Department of a large quaternary hospital between March 8 and June 3, 2020. We built logistic regression models to analyze how race, ethnicity, area deprivation index, English as a primary language, homelessness, and illicit substance use impacted the likelihood of admission while controlling for disease severity and timing of admission in relation to the start of data collection. We had 1302 recorded Emergency Department visits of patients diagnosed with SARS-CoV-2. White, Hispanic, and African American patients made up 39.2%, 37.5%, and 10.4% of the population respectively. Primary language was recorded as English for 41.2% and non-English for 30% of patients. Among the social determinants of health assessed, we found that illicit drug use significantly increased the likelihood for admission to the medical ward (odds ratio 4.4, confidence interval 1.1–17.1, *P* = .04), and that having a language other than English as a primary language significantly increased the likelihood of ICU admission (odds ratio 2.6, confidence interval 1.2–5.7, *P* = .02). Illicit drug use was associated with an increased likelihood of medical ward admission, potentially due to clinician concerns for complicated withdrawal or blood-stream infections from intravenous drug use. The increased likelihood of ICU admission associated with a primary language other than English may have been driven by communication difficulties or differences in disease severity that our model did not detect. Further work is required to better understand drivers of disparities in hospital COVID-19 care.

## 1. Introduction

The COVID-19 pandemic has highlighted significant racial and socioeconomic disparities in COVID-19-related outcomes.^[1–3]^ Additionally, studies have shown worse COVID-19 outcomes in correlation with other social determinants of health including mental illness, homelessness, substance use, and primary language other than English.^[2,4–6]^ Better understanding of these disparities and means to address them are not only a moral imperative but also critical to adequately confronting the ongoing COVID-19 pandemic and future pandemic disease.

There are many known and well-supported drivers of disparities in COVID-19 outcomes, many of which are structural issues.^[7]^ Such issues include lack of health insurance coverage and thus poor or delayed access to care,^[3,8]^ increased exposure through household crowding and working “essential jobs” with inadequate access to personal protective equipment,^[9]^ and segregated neighborhoods with less access to health services.^[10,11]^ Additionally, increased incidence and severity of symptomatic COVID-19 are also driven by differences in medical comorbidities, such as hypertension, diabetes, and obesity, that have higher prevalence among Black and Latinx populations.^[12]^

In addition to known structural issues, differences in hospital-based care may be contributing to disparities in outcomes.^[13]^ However, results have been mixed and It is unclear what might be driving such in-hospital differences.^[14–16]^ Implicit bias and explicit biases in clinical decision making – for example in clinical protocols – have been raised as possible drivers of disparities in care.^[17–19]^ A particularly important point in the COVID-19 care cascade is hospital admission. Hospital admission decisions can vary widely across providers and hospitals,^[20,21]^ with studies outside of COVID-19 care showing differences in hospital admissions along racial and socioeconomic lines.^[22,23]^ To date, few studies have looked at how multiple social determinants of health might impact hospital admission decisions during the COVID-19 pandemic. This study looks at how race, ethnicity, and other social determinants of health correlate with admission to both the medical ward and intensive care unit (ICU).

## 2. Methods

### 2.1. Setting

This study took place at massachusetts general hospital (MGH) in Boston, MA between March 8 and June 3, 2020. MGH is a 999-bed quaternary teaching hospital.

### 2.2. Study design and data collection

We conducted a retrospective analysis using the MGH COVID-19 Data Registry, which includes all patients confirmed to have SARS-CoV-2-infected who presented to the MGH emergency department (ED). The database was compiled with both data extraction from the electronic medical record as well as manual chart reviews. Trained reviewers collected demographics, comorbid conditions, medications, and epidemiological risk factors for SARS-CoV-2. Patient data was collected from each day of their hospital stay, starting from presentation to the ED; each patient had 28 days of follow-up from the date of presentation to evaluate for mortality.

The area deprivation index (ADI) scores for Massachusetts were collected on July 28, 2020 from Health Services Advisory Group website.^[24]^ The ADI is composed of 17 education, employment, housing-quality, and poverty measures drawn from both the National Census and American Community Survey data,^[25]^ and provides a disparity score by 9-digit zip code.^[26]^ The ADI is scored out of 10 and is inversely related to socioeconomic status (i.e., 10 indicating the lowest socioeconomic status). Because our database only included patients 5-digit zip codes, we averaged ADI scores within each 5-digit zip code to provide a score for each patient.

### 2.3. Participants

We included all patients 18 years and older who presented to the MGH ED between March 8 and June 3, 2020 who had SARS-CoV-2 infection confirmed via polymerase-chain reaction nasopharyngeal swab testing.

### 2.4. Analysis

Participant characteristics were summarized descriptively. Comparisons between patients discharged home, admitted to the medical ward, or admitted directly to the ICU were made with Wilcoxson rank sum and Pearson chi-square tests for continuous and categorical variables, respectively. Impact of timing during the pandemic was assessed as days since data collection started (March 8, 2020).

All tests were 2-sided and a *P* value < .05 was considered statistically significant. All variables were initially assessed for significance using univariable analysis comparing: Patients discharged home versus admitted to the medical ward and; Patients admitted to the medical ward versus ICU (see Tables S1 and S2, Supplemental Digital Content, http://links.lww.com/MD/I601, which shows the results of univariable analysis). A Multivariable logistic regression was fitted separately comparing: Patients discharged home versus admitted to the medical ward and; Patients admitted to the medical ward versus ICU. We opted for 2 logistic regression models to reflect the distinct clinical decision making processes in the ED (i.e., “discharge home” vs “admit to medical ward,” and “admit to medical ward” vs “admit to ICU”).”

Our key associations of interest were race, ethnicity, ADI, English as a primary language, homelessness, and illicit substance use (opiates, cocaine, methamphetamine); variables also included age, gender, and clinical comorbidities, including body mass index (mg/kg^2^) and clinical severity. We evaluated disease severity using clinical severity scores (sequential organ failure assessment, Charlson comorbidity index) and laboratory markers found in other risk severity scores,^[27,28]^ specifically, C-reactive protein (mg/L), ferritin (ug/L), D-dimer (ng/mL), creatine kinase (U/L), troponin (ng/L), procalcitonin (ng/mL), absolute lymphocyte count (K/mL), and blood urea nitrogen (mg/dL). Timing of admission was calculated as days after the first date of data collection (March 8, 2020). In our regression, we controlled for timing of admission and included the square of timing of admission to evaluate how the effect changed over time. To build our regression models, we first included a priori variables based on clinical understanding (i.e., age, sex, sequential organ failure assessment, C-reactive protein, ferritin, and troponin), and then added variables that were significant on univariable analysis.” Variables were excluded if they showed significant co-linearity (variance inflation factors over 10). We used stepwise, backward selection for our logistic regression model, using a *P* value of over 0.2 as a cutoff to remove variables. Potential interaction between significant variables was explored.

Additionally, we divided differences in number of admissions in 3 groups to visually evaluate changes in admission over time. Groups were created as general phases of the surge in SARS-CoV-2 admissions in our hospital, representing changes in comfort with diagnosis and clinical management of COVID-19. Changes in admission patterns over time were assessed using the Jonckheere–Terpstra test for trend. All data were analyzed using Stata Statistical Software (Release 16. College Station, TX: StataCorp LLC).

### 2.5. Ethical approval

Study approval was obtained from the Mass General Brigham Institutional Review Board (2020P001789).

## 3. Results

Overall, 1302 visits were recorded of patients who presented to the ED and tested positive for SARS-CoV-2 between March 8 and June 3, 2020 (Table 1). The mean age was 59 years (standard deviation 18), and 560 (42.0%) were women. Medical comorbidities were present in 1162 (89.3%) patients. 807 (62.0%) patients had a body mass index > or equal to 30, 649 (49.8%) had hypertension, 425 (32.6%) had diabetes, 383 (29.4%) with lung disease, 219 (16.8%) had chronic kidney disease, and 19 (1.5%) had human immunodeficiency virus.

**Table 1:** Baseline characteristics. Descriptive statistics of the patient cohort.

	Overall	Discharged home	Admitted to medical ward	Admitted to ICU	*P* value
Total, N (%)	1302	302 (22.0)	814 (59.4)	186 (13.6)	< .001
Age, mean, yr (SD)	59 (18)	55 (16)	60 (19)	62 (16)	< .001
Female sex, N (%)	560 (43.0)	148 (49.0)	354 (43.5)	58 (31.2)	.001
Race/Ethnicity N (%)					
White	510 (39.2)	98 (32.5)	351 (43.1)	61 (32.8)	.003
Black	136 (10.4)	24 (7.9)	93 (11.4)	19 (10.2)	.232
Asian	52 (4.0)	13 (4.3)	31 (3.8)	8 (4.3)	.932
Hispanic	465 (35.7)	134 (44.4)	266 (32.7)	65 (34.9)	.001
Other	29 (2.2)	11 (3.6)	15 (1.8)	3 (1.6)	< .001
Missing	110 (8.5)	22 (7.3)	58 (7.1)	30 (16.1)	< .001
Comorbidities, N (%)					
Hypertension	649 (49.8)	132 (43.7)	420 (51.6)	97 (52.2)	.051
Diabetes	425 (32.6)	80 (26.5)	269 (33.0)	76 (40.9)	.004
Lung disease	383 (29.4)	91 (30.1)	246 (30.2)	46 (24.7)	.547
Kidney disease	219 (16.8)	28 (9.3)	157 (19.3)	34 (18.3)	< .001
HIV	19 (1.5)	3 (1.0)	13 (1.6)	3 (1.6)	.743
BMI ≥ 30mg/kg^2^	807 (62.0)	184 (60.9)	488 (60.0)	135 (72.6)	.005
Homeless, N (%)	40 (3.1)	5 (1.7)	33 (4.1)	2 (1.1)	.028
Illicit drug use, N (%)	62 (4.8)	5 (1.7)	51 (6.3)	6 (3.2)	.003
Primary language, N (%)					
English	536 (41.2)	130 (43.0)	346 (42.5)	60 (32.3)	.001
Non-English	391 (30.0)	117 (38.7)	211 (25.9)	63 (33.9)	< .001
Missing	375 (28.8)	55 (18.2)	257 (31.6)	63 (33.9)	< .001
ADI, mean (SD)	6.0 (0.1)	6.2 (0.3)	5.9 (0.1)	6.2 (0.2)	.722

ICU = intensive care unit, SD = standard deviation, HIV = human immunodeficiency virus, BMI = body mass index, ADI = area deprivation index.

White patients accounted for 510 patients (39.2%), with Hispanic and African American patients comprising the second and third largest populations at 465 (37.5%) and 136 (10.4%), respectively. The primary language was recorded as English for 536 patients (41.2%) and nonEnglish for 391 (30%) patients. Missing data was common with 8.5% of participants missing race data and 28.8% missing language data. The mean ADI for the population was 6.0 (standard deviation 0.1). There were 40 patients (3.1%) documented as homeless, and 62 patients (4.8%) reported using illicit substances (i.e., opiates, methamphetamine, cocaine).

**Figure 1.**
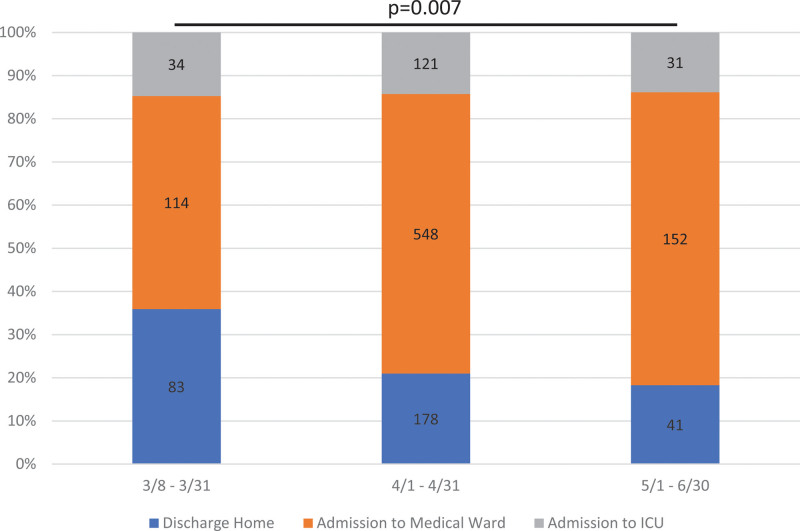
Changes in admission patterns over time. Comparing outcomes of patients with COVID-19 from the Emergency Department. Outcomes included discharge home, admission to the medical ward, and admission to the ICU. Dates were selected by phases of the surge in cases of patients with COVID-19. Numbers in each column represent the number of patients with each outcome. In comparing the trend over the 3 time periods, the change in admitting patterns over time were significant (*P* = .007). ICU = intensive care unit.

Most patients, 814 (59.4%), were admitted to the medical ward with 302 (22.0%) being sent home and 186 (13.6%) admitted to the ICU (Table 1). Admission patterns changed significantly over the course of the collected data (*P* = .007) (Fig. 1). We found the proportion of patients admitted to the medicine ward increased over this time period (54.1%–68.2%), while the proportion of patients discharged home decreased from 31.3% to 20.1% and the percentage of patients directly admitted from the ED to the ICU was relatively constant (14.6%–11.8%, *P* = .187).

In a univariable analysis, social determinants of health that were associated with admission to the medical ward versus discharge to home was illicit drug use with an odds ratio (OR) of 4.0 (*P* = .004), while a non-English primary language with an OR of 0.7 (*P* = .01), nonwhite race with an OR of 0.6 (*P* = .001) and ADI with an OR of 0.9 (*P* = .04) were negative predictors of admission (Table 2). In Multivariable analysis, illicit drug use was positively associated with medical ward admission with an OR of 4.4 and its 95% confidence interval 1.1 to 17.1 (*P* = .04). Timing of admission in days was also significantly associated with admission to the medical ward with an OR of 1.1 (*P* < .001) (see Table S3a, Supplemental Digital Content, http://links.lww.com/MD/I602, which shows the univariable and Multivariable results for timing of admission analysis). The square of the admission date variable was negatively correlated with admission with an OR of 0.999 (*P* = .002), and thus the effect of date of admission waned over time.

**Table 2:** Social determinants of health associated with admission to medical ward versus discharge home. Logistic regression findings for our key associations for patients admitted to the medical ward rather than discharged home from the emergency department.

	Univariable analysis		Multivariable analysis	
	Odds ratio (95% CI)	*P* value	Odds ratio (95% CI)	*P* value
Nonwhite race (reference: white race)	0.6 (0.5, 0.8)	.001	1.5 (0.9, 2.6)	.10
ADI (per unit in the score)	0.9 (0.9, 1.0)	.04	0.9 (0.8, 1.0)	.19
Non-English primary language (reference: English)	0.7 (0.5, 0.9)	.01	1.0 (0.6, 1.6)	.98
Homelessness (reference: housed)	2.5 (1.0, 6.5)	.06	0.7 (0.2, 2.5)	.54
Illicit drug use (reference: no illicit drug use)	4.0 (1.6, 10.1)	.004	4.4 (1.1, 17.1)	.04

The regression model controlled for age, sex, C-reactive protein (CRP), blood urea nitrogen (BUN), absolute lymphocyte count (ALC), ferritin, hypotension on hospital admission, oxygen requirement on hospital admission, history of renal disease, timing of admission; Illicit drug use included opiates, cocaine, methamphetamine use.

ADI = area deprivation index, CI = confidence interval.

In univariable analysis, when comparing factors associated with admission to the ICU rather than admission to the medicine ward included, we found that nonwhite race with an OR of 1.3 (*P* = .01), each unit increase in ADI with an OR of 1.1 (*P* = .04), and non-English primary language with an OR of 1.7 (*P* = .01) were positively associated with ICU admission (Table 3). In multivariable analysis, a primary language other than English with an OR of 2.6 and 95% confidence interval of 1.2 to 5.7 (*P* = .02) was significantly associated with ICU admission over admission to the medical ward. No interaction was seen between race and ADI. Timing of admission in days was significantly associated with admission to the ICU with an OR of 0.9 (*P* = .01) (see Table S3b, Supplemental Digital Content, http://links.lww.com/MD/I603, which shows the univariable and Multivariable results for timing of admission analysis).

**Table 3:** Social determinants of health associated with admission to ICU versus medical ward. Logistic regression findings for our key associations for patients admitted to the Intensive Care Unit rather than admitted to the medical ward.

	Univariable analysis		Multivariable analysis	
	Odds ratio (95% CI)	*P* value	Odds ratio (95% CI)	*P* value
Nonwhite race (reference: white race)	1.4 (1.0, 1.9)	.10	1.0 (0.4, 2.2)	.99
ADI (per unit in the score)	1.1 (1.0, 1.2)	.04	1.0 (0.8, 1.1)	.58
Non-English primary language (reference: English)	1.7 (1.2, 2.6)	.01	2.6 (1.2, 5.7)	.02
Homelessness (reference: domiciled)	0.3 (0.1, 1.1)	.06	1.0 (0.1, 9.8)	.98
Illicit drug use (reference: no illicit drug use)	0.5 (0.2, 1.2)	.11	0.7 (0.2, 3.6)	.71

The regression model controlled for age, sex, C-reactive protein (CRP), creatinine kinase (CK), D-dimer, troponin, ferritin, oxygen requirement on hospital admission, body mass index (BMI) ≥ 30mg/kg^2^, timing of admission; Illicit drug use included opiates, cocaine, methamphetamine use.

ADI = area deprivation index, CI = confidence interval, ICU = intensive care unit.

## 4. Discussion

When looking at social determinants of health and controlling for age, sex, medical comorbidities, and clinical severity, we found that illicit drug use was associated with admission to the medical ward versus discharging home, and that not having English as a primary language was associated with admission to the ICU. We found that admission decisions did not differ by race, ethnicity, Absolute Disparity Index, or homelessness. We also found that timing of admission in relation to the start of data collection was significantly associated the decision to admit to the medical ward versus discharge home, though was not associated with the decision to admit to the ICU versus the medical ward.

Our finding that illicit drug use was independently associated with admission to the medical ward is in line with population-based studies, where substance use was associated with more severe disease and hospitalization.^[29–32]^ However, the findings of these population-based studies may have been related to delays in presentation to care as these studies did not control for disease severity at hospital admission. Patients with illicit drug use may have been preferentially admitted due to intoxication or out of concern for complicated withdrawal, supported by data showing that nonfatal opioid overdoses increased significantly during the pandemic.^[33]^ Our dataset does not include which patients were seeking medical care for withdrawal or initiation of opioid agonist substitution treatment concurrent with COVID-19 infection, which may also have increased admission likelihood. It is also possible that our finding reflected concern for other causes of fevers and malaise in this group of patients. Particularly for patients with a history of intravenous drug use, the desire to also rule out blood-stream infections may have led to increased likelihood of admission. Finally, outpatient services were disrupted for patients with illicit drug use during the pandemic, and this may have represented clinicians more conservative approach if follow-up care was not in place.^[34,35]^

It is not immediately clear why not having English as a primary language was a factor dictating ICU admission over medical ward admission. We did have significant missing data on primary language, and patients in the ICU were twice as likely to have race data missing. Thus, this finding may represent availability bias. However, challenges in patient-provider communication have been shown to drive disparities in hospital admission patterns by primary language.^[36]^ While we did not have data on providers primary language or the use of interpreter services in our cohort, communication difficulties may have been more challenging among the very sick patients who were considered for ICU admission. Additionally, end-of-life discussions that led to patient deferral of ICU admission may have been made more difficult by language barriers.^[37]^ Finally, among COVID-19 patients in Massachusetts, primary language has been shown to be significantly associated with disease severity.^[38]^ Our finding may therefore be due to increased disease severity among patients who did not have English as a primary language that our model was unable to detect.

A strength of this study is that it is among the first to look at how admission decisions in the Emergency Department might vary by social determinants of health. Previous studies have looked at factors influencing admission on a population level or focused predominantly on clinical data.^[39–41]^ Other studies evaluating hospital or ICU admission decisions for COVID-19 patients by race and ethnicity have largely found no differences, which supports our findings in this study.^[42,43]^ However, a study done by Russell et al^[44]^ looked at hospital admissions from an Emergency Department observation unit and found patients of Hispanic ethnicity were more likely to be admitted to the hospital when controlling for clinical factors. It is unclear why hospital admission from an observation unit was different, though may have related to differential admission to the observation unit initially, or possibly the communication issues noted above.

Another strength of this study is the ADI, which enables evaluation social determinants based on a patient’s zip code. The ADI has been used in previous studies in the US and found that severity of COVID-19 was more severe by race and independent of ADI,^[38]^ though no studies to date have evaluated the association of ADI with hospital-based outcomes. We also included markers of disease severity that have been highlighted in the literature or used in disease severity models to provide a marker of disease severity. Given that decisions for hospital admission may not be entirely objective and may depend on a provider’s perception of disease severity, hospital bed availability, and likelihood of outpatient follow-up among other issues, controlling for disease severity was critical to evaluate differences in admission patterns by social determinants. Controlling for disease severity was also important to avoid sampling bias because prior work had showed that Hispanic patients presenting to our hospital were younger with fewer comorbidities.^[45]^

The major limitations of this study were the retrospective study design at just 1 hospital, a relatively small number of patients, and focus on 1 wave during the ongoing COVID-19 pandemic. We also did not have significantly detailed data on psychiatric illness and types of substance use and thus were limited in our analysis of these social determinants of health in this study. Additionally, our dataset only included patients who presented to the hospital’s Emergency Department, limiting the scope of the study to highlight other systematic disparities in the cascade of COVID-19 care.

In summary, we found that the decision to admit patients to the medical ward versus discharging them home was independently associated with illicit drug use. Additionally, we found that the decision to admit patients to the ICU versus medical ward was associated with having a primary language other than English. Admission decisions were not associated with race, ethnicity, ADI, or homelessness. More work should focus on ensuring improved communication and translation services for critically ill patients in the emergency department. additionally, as we strive to ensure equitable care for our patients, more research should delve more deeply into differences in decision making at various points in the care cascade to see how they differ by social determinants of health.

## Acknowledgments

The authors would like to thank the time and effort of the dedicated manual chart reviewers and data managers from the MGH COVID registry who made this work possible. This study was unfunded. JEH reports consulting fees from Merck and stock ownership in Natera.

## Author contributions

**Conceptualization:** Peter K. Olds, Benjamin P. Geisler, Jessica E. Haberer.

**Formal analysis:** Peter K. Olds, Nicholas Musinguzi, Benjamin P. Geisler, Jessica E. Haberer.

**Methodology:** Peter K. Olds, Benjamin P. Geisler.

**Software:** Peter K. Olds, Jessica E. Haberer.

**Supervision:** Jessica E. Haberer.

**Writing – original draft:** Peter K. Olds.

**Writing – review & editing:** Peter K. Olds, Benjamin P. Geisler, Jessica E. Haberer.

## Supplementary Material






