# Supplementary material for: Evaluating disparities by social determinants in hospital admission decisions for patients with COVID-19 quaternary hospital early in the pandemic
Source: Medicine (Baltimore). 2023 Mar 10;102(10):e33178. doi: 10.1097/MD.0000000000033178 (PMC9997198; doi:10.1097/MD.0000000000033178)
Supplement: Supplementary file 1 [file medi-102-e33178-s001.pdf]

Supplemental Table 1: Univariable analysis of criteria associated with admission to Medical Ward versus Discharge Home. Using an initial cut-off of p=0.10, variables used at the start of building the multivariable analysis are highlighted in blue. CI: Confidence Interval; CRP: C-reactive protein; BUN: Blood urea nitrogen; ALC: Absolute lymphocyte count; CK: Creatinine kinase; SOFA: Sequential Organ Failure Assessment; CCI: Charlson Comorbidity Index; HIV: Human immunodeficiency virus; ADI: Area Deprivation Index

Variable	OR (95% CI)	p value
**Age, years (per year)**	1.0 (1.0-1.0)	<0.001
**Female Sex (reference: male sex)**	0.8 (0.6-1.0)	0.09
**CRP, mg/L (per unit)**	1.0 (1.0-1.0)	<0.001
**BUN, mg/dL (per unit)**	1.0 (1.0-1.1)	<0.001
**ALC, K/uL (per unit)**	0.8 (0.7-1.0)	0.09
**CK, U/L (per unit)**	1.0 (1.0-1.0)	0.09
**D-dimer, ng/mL (per unit)**	1.0 (1.0-1.0)	0.27
**Procalcitonin, ng/mL (per unit)**	1.7 (1.0-1.2)	0.20
**Troponin, ng/L (per unit)**	1.0 (1.0-1.0)	<0.001
**Ferritin, ug/L (per unit)**	1.0 (1.0-1.0)	0.004
**SOFA (per unit in the score)**	1.0 (0.9-1.0)	0.41
**CCI (per unit in the score)**	1.2 (1.1-1.3)	<0.001
**Hypotension (SBP <90mmHg) on admission (reference: no hypotension on admission)**	4.3 (1.3-14.3)	0.02
**Oxygen requirement on admission (reference: no oxygen requirement on admission)**	5.4 (3.6-8.1)	<0.001
**History of renal disease (reference: no history)**	2.4 (1.4-3.6)	<0.001
**History of lung disease (reference: no history)**	1.0 (0.7-1.3)	0.94
**History of hypertension (reference: no history)**	1.4 (1.1-1.8)	0.02
**History of diabetes (reference: no history)**	1.4 (1.0-1.8)	0.04
**History of congestive heart failure (reference: no history)**	3.1 (1.8-5.4)	<0.001
**History of stroke (reference: no history)**	2.7 (1.3-5.5)	0.01
**History of HIV (reference: no history)**	1.6 (0.5-5.7)	0.45
**BMI ≥30mg/kg^2 (reference: BMI <30mg/kg^2)**	1.0 (0.7-1.3)	0.72
**Non-white race (reference: White race)**	0.6 (0.5-0.8)	0.001
**Area Deprivation Index (ADI)**	0.9 (0.9-1.0)	0.04
**Non-English primary language (reference: English)**	0.7 (0.5-0.9)	0.01
**Homelessness (reference: domiciled)**	2.5 (1.0-6.5)	0.06
**Illicit drug use (opiates, cocaine, methamphetamines) (reference: no illicit drug use)**	4.0 (1.6-10.1)	0.004
**Current cigarette smoker (reference: no active cigarette use)**	2.3 (1.2-4.5)	0.01

Supplemental Table 2: Univariable analysis of criteria associated with admission to ICU versus Medical Ward Using an initial cut-off of p=0.10, variables used at the start of building the multivariable analysis are highlighted in blue. CI: Confidence Interval; CRP: C-reactive protein; BUN: Blood urea nitrogen; ALC: Absolute lymphocyte count; CK: Creatinine kinase; SOFA: Sequential Organ Failure Assessment; CCI: Charlson Comorbidity Index; HIV: Human immunodeficiency virus; ADI: Area Deprivation Index

	OR (95% CI)	p value
**Age, years (per year)**	1.0 (1.0-1.0)	0.15
**Female Sex (reference: male sex)**	0.6 (0.4-0.8)	0.002
**CRP, mg/L (per unit)**	1.0 (1.0-1.0)	<0.001
**BUN, mg/dL (per unit)**	1.0 (1.0-1.0)	<0.001
**ALC, K/uL (per unit)**	0.9 (0.6-1.2)	0.31
**CK, U/L (per unit)**	1.0 (1.0-1.0)	0.01
**D-dimer, ng/mL (per unit)**	1.0 (1.0-1.0)	<0.001
**Procalcitonin, ng/mL (per unit)**	1.0 (1.0-1.1)	0.03
**Troponin, ng/L (per unit)**	1.0 (1.0-1.0)	0.002
**Ferritin, ug/L (per unit)**	1.0 (1.0-1.0)	<0.001
**SOFA (per unit in the score)**	1.1 (1.0-1.2)	0.003
**CCI (per unit in the score)**	1.0 (1.0-1.1)	0.66
**Hypotension (SBP <90mmHg) on admission (reference: no hypotension on admission)**	2.3 (1.3-4.2)	0.01
**Oxygen requirement on admission (reference: no oxygen requirement on admission)**	6.2 (4.2-9.3)	<0.001
**History of renal disease (reference: no history)**	1.0 (0.7-1.5)	0.99
**History of lung disease (reference: no history)**	0.8 (0.6-1.2)	0.32
**History of hypertension (reference: no history)**	1.0 (0.7-1.4)	0.88
**History of diabetes (reference: no history)**	1.4 (1.0-1.9)	0.04
**History of congestive heart failure (reference: no history)**	0.7 (0.4-1.1)	0.10
**History of stroke (reference: no history)**	0.7 (0.4-1.4)	0.29
**History of HIV (reference: no history)**	1.0 (0.3-3.6)	0.99
**BMI ≥30mg/kg^2 (reference: BMI <30mg/kg^2)**	1.8 (1.2-2.5)	0.002
**Non-white race (reference: White race)**	1.4 (1.0-1.9)	0.10
**Area Deprivation Index (ADI)**	1.1 (1.0-1.2)	0.04
**Non-English primary language (reference: English)**	1.7 (1.2-2.6)	0.01
**Homelessness (reference: domiciled)**	0.3 (0.1-1.1)	0.06
**Illicit drug use (opiates, cocaine, methamphetamines) (reference: no illicit drug use)**	0.5 (0.2-1.2)	0.11
**Current cigarette smoker (reference: no active cigarette use)**	0.8 (0.4-1.5)	0.45
