# Supplementary material for: Evaluating disparities by social determinants in hospital admission decisions for patients with COVID-19 quaternary hospital early in the pandemic
Source: Medicine (Baltimore). 2023 Mar 10;102(10):e33178. doi: 10.1097/MD.0000000000033178 (PMC9997198; doi:10.1097/MD.0000000000033178)
Supplement: Supplementary file 2 [file medi-102-e33178-s002.pdf]

Supplemental table 3a: Multivariate findings regression findings for date of admission for admission to the Medical Ward rather than discharged home from the Emergency Department.* CI: Confidence Interval.

	**Univariate Findings**		**Multivariate Findings**	
	**Odds Ratio (95% CI)**	**p-value**	**Odds Ratio (95% CI)**	**p-value**
**Date of admission, days (per day)**	1.0 (1.0, 1.0)	<0.001	1.1 (1.0, 1.2)	<0.001
**Date of admission squared (per unit)**	1.0 (1.0, 1.0)	<0.001	0.999 (0.999, 1.000)	0.002

*The regression model controlled for age, sex, C-reactive protein (CRP), blood urea nitrogen (BUN), absolute lymphocyte count (ALC), ferritin, hypotension on hospital admission, oxygen requirement on hospital admission, history of renal disease, race, Area Disparity Index (ADI), homelessness, illicit drug use.

Supplemental table 3b: Multivariate findings regression findings for date of admission for admission to the Medical Ward rather than discharged home from the Emergency Department.* CI: Confidence Interval.

	**Univariate Findings**		**Multivariate Findings**	
	**Odds Ratio (95% CI)**	**p-value**	**Odds Ratio (95% CI)**	**p-value**
**Timing of admission, days (per day)**	1.0 (1.0, 1.0)	0.04	0.9 (0.8, 1.0)	0.01
**Timing of admission squared (per unit)**	1.0 (1.0, 1.0)	0.05	1.001 (1.000, 1.002)	0.01

*The regression model controlled for age, sex, C-reactive protein (CRP), creatinine kinase (CK), D-dimer, troponin, ferritin, oxygen requirement on hospital admission, ≥30mg/kg^2, race, area disparity index (ADI), homelessness, illicit drug use.
