# Supplementary material for: Evaluating disparities by social determinants in hospital admission decisions for patients with COVID-19 quaternary hospital early in the pandemic
Source: Medicine (Baltimore). 2023 Mar 10;102(10):e33178. doi: 10.1097/MD.0000000000033178 (PMC9997198; doi:10.1097/MD.0000000000033178)
Supplement: Supplementary file 3 [file medi-102-e33178-s003.pdf]

Supplemental table 3b: Multivariable findings regression findings for date of admission for admission to the Medical Ward rather than discharged home from the Emergency Department.* CI: Confidence Interval.

	Univariable Findings		Multivariable Findings	
	**Odds Ratio (95% CI)**	**p-value**	**Odds Ratio (95% CI)**	**p-value**
**Timing of admission, days (per day)**	1.0 (1.0, 1.0)	0.04	0.9 (0.8, 1.0)	0.01
**Timing of admission squared (per unit)**	1.0 (1.0, 1.0)	0.05	1.001 (1.000, 1.002)	0.01

*The regression model controlled for age, sex, C-reactive protein (CRP), creatinine kinase (CK), D-dimer, troponin, ferritin, oxygen requirement on hospital admission, body mass index (BMI) $\geq 30mg/kg^2$, race, area disparity index (ADI), homelessness, illicit drug use.
